# The Effect of Significant International Sports Events on Qualified Detoxification Treatment Outcome - Do Drop-Out Rates Change during UEFA European Championship?

**DOI:** 10.1371/journal.pone.0167446

**Published:** 2016-12-28

**Authors:** Yvonne Sofin, Heidi Danker-Hopfe, Peter Neu

**Affiliations:** 1 Jewish Hospital Berlin - Clinic for Psychiatry and Psychotherapy, Berlin, Germany; 2 Competence Center for Sleep Medicine, Charité - School of Medicine, Campus Benjamin Franklin, Berlin, Germany; 3 Charité - School of Medicine, Campus Benjamin Franklin, Berlin, Germany; Universita Campus Bio-Medico di Roma, ITALY

## Abstract

No previous studies have evaluated the influence of significant international sports events on qualified detoxification treatment outcome. This prospective study examines the impact of the 2012 UEFA European Football Championship on inpatient treatment outcome of alcohol dependent patients. Hospital admission and premature drop-out rates of consecutively admitted alcohol dependent patients were determined before, during and immediately after the UEFA Championship in the year 2012. The admission rate of male patients increased significantly after the European Football Championship had ended whereas for female patients, no change in admission rate was found. Daily average discharge rate was calculated. No statistically relevant differences between the treatment days before, during and after the UEFA Championship was found for the discharges. During the tournament, exclusively male patients dropped out. Our results are consistent with an interpretation of an association between European Football Championship and detoxification treatment outcome. Further research to replicate and extend our findings is necessary.

## Introduction

Soccer is one of the most popular spectator sports worldwide. The UEFA European Football Championship is an international football tournament contested every four years and is considered the second most prestigious tournament after the FIFA World Cup. From 8th of June to 1st of July 2012, the 14th European Football Championships was hosted by Poland and Ukraine.

Alcohol is strongly associated with popular sport events [1]. That is why sport is saturated by the promotion of alcohol. The linkage of alcohol marketing and professional televised sports is widely present and known to effect alcohol consumption [2]. In the light of the above, it is not surprising that the Danish brewery Carlsberg was one of the main sponsors of the 2012 European Football Championships.

No previous studies have evaluated the influence of significant international sports events on qualified detoxification treatment (QDT) outcome. The aim of the present study was to investigate whether drop-out rates of consecutively admitted alcohol dependent patients in a psychiatric hospital in Germany changed during UEFA European Championship. We analyzed the hospital admission and prematurely discharge rates before, during and immediately after the 2012 UEFA Championshipin to explore whether admission and discharge rates changed prior, during and after the European Football Championships and to identify whether the occurrence of popular spectator sports may serve as predictor for treatment outcome during inpatient detoxification treatment.

## Methods

### Participants

In 2012, 125 consecutively admitted alcohol dependent patients were included in the study. All patients fulfilled the DSM-IV criteria for alcohol addiction. Exclusion criterion was non-capacity of giving informed consent (e.g. due to severe organic or psychiatric disorders like Korsakow syndrome). For diagnosis of addiction and concomitant diseases Diagnostic and Statistical Manual (DSM) edition IV was applied.

### Setting and treatment procedure

This prospective study was conducted on two specialized inpatient units for qualified detoxification treatment of addiction diseases in a psychiatric hospital in Berlin, Germany. Our research as well as the used informed consent form were approved by the Ethics Commission of the Charité –Universitätsmedizin Berlin. All participants provided their written consent to participate in this study.

The treating team comprised medical doctors, psychologists, specialized nurses, occupational therapists, physiotherapists and social workers. The qualified detoxification treatment consisted of three steps. While detoxification the patients were withdrawn from alcohol and, where needed, withdrawal symptoms were treated. In the second step, the patients had to attend to at least ten group-therapy sessions and five psycho-educational group-sessions. In the third step, the preparation of transition to a long-term follow-up treatment after hospital discharge including the attendance of five self-help groups outside the clinic was conducted. The average treatment took between 12 and 16 days, but could last longer in case of persisting withdrawal symptoms or particularly severe general condition. Clomethiazole at tapered doses was used for alcohol detoxification. The severity of alcohol withdrawal symptoms was captured according to the CIWA Withdrawal Score [3]. All patients were admitted electively for qualified detoxification treatment except for emergency admissions.

### Definition of outcome criteria

The treatment was considered successfully completed if the patient remained abstinent while hospital stay and participated in the treatment program as described above until regular discharge. The attendance to at least ten group-therapy sessions, five psycho-educational group-sessions and additional five self-help groups outside the clinic was mandatory.

The treatment was considered aborted if the patient left against medical advice or due to disciplinary early discharge. Substance use or refusal to participate in the treatment program led to disciplinary discharge.

### Data analysis

**Fig 1:**
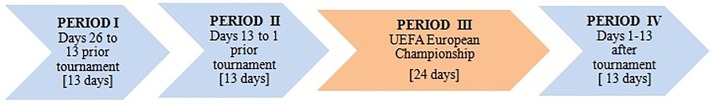
Examined periods ahead during and after UEFA European Championship 2012.

Four periods were defined to differentiate patients at risk to prematurely terminate treatment due to the UEFA European Championship from those patients who dropped out independently from the Championship. All patients received an elaborate description on the average treatment duration in preparation for the qualified detoxification treatment. As the average detoxification treatment took 14 days, we defined periods of 13 days. Period I covered the days 26 to 13 ahead to the UEFA European Championship 2012. Period II covered day 13 to day 0 ahead the Championship. Period III covered the tournament (24 days) while period IV covered the 13 days after termination of the Championship. The patients admitted in periods 2 and 3 were therefore considered at risk to miss tournament days. Patients admitted in periods 1 and 4 were considered as non-jeopardized patients as they were able to finish treatment regularly prior to UEFA European Championship start, respectively after the tournament (Fig 1).

For further elaboration, two patient groups were considered: The first group comprised of all patients that were admitted for qualified detoxification treatment during the four periods. The second group consisted of all patients that were discharged during the defined periods.

Medical anamnesis was captured by an experienced physician during structured face to face admission interview. Statistical analyses were carried out using SAS (statistical analysis system) software by SAS Institute. The examination was carried out with log likelihood qui square test. Tests on group differences were examined with a two-sided significance level of p <0.05. Age differences were examined with Wilcoxon 2 sample test. P values of 0.05 or less were considered statistically significant.

## Results

### Patient characteristics

The group of admitted patients comprised of 125 patients that could be included in the study. 93 (74.4%) patients were male, 32 (25.6%) were female. The mean age was 45.5 (±11.9) years. The patient characteristics are shown in Table 1.

**Table 1:** Patient characteristics of all admitted patients.

Characteristic	Mean age [years] ± SD	p value
Total (N = 125)	45.5 ± 11.9	
Male sex (N = 93)	44.2 ± 11.9	0.4546
Female sex (N = 32)	45.9 ± 11.9	
Treatment completed (N = 93)	45.4 ± 12.2	0.6760
Dropped out of treatment (N = 32)	45.8 ± 11.2	

Overall, 93 (74.4%) of the patients admitted during the four periods, completed detoxification treatment. The individuals that dropped out comprised of 25 (26.9%) male patients that terminated the treatment irregularly while only 7 (21.9%) of the female patients dropped out. We found no statistically significant differences (p = 0.4546) between the male patient's mean age (44.2 ± 11.9) and the female patient's mean age (45.9 ± 11.9). Neither we found significant differences in the mean age of patients that completed the treatment regularly and the patients that dropped-out of treatment (p = 0.6760). In our study, we further found no significant differences in the distribution of treatment completers and drop-outs between the sexes (p = 0.5712).

Table 2 shows the patient characteristics of the second group which consisted of all patients that were discharged during the four periods.

**Table 2:** Patient characteristics (discharges).

Characteristic	Mean age [years] ± SD	p value
Total (N = 116)	46.5 ± 11.9	
Treatment completed (N = 81)	47.1 ± 12.4	0.6387
Dropped out of treatment (N = 35)	45.2 ± 10.9	

81 (69.8%) of the patients discharged during the four periods, completed detoxification treatment regularly, while 35 (30.2%) dropped out. The individuals that dropped out comprised of 28 (35.9%) male patients that terminated the treatment irregularly while 7 (18.4%) of the female patients dropped out. As before for the admissions, we found no significant difference in the mean age of patients that completed the treatment regularly and the patients that dropped-out of treatment (p = 0.6387).

### European Football Championship 2012

Admission as well as prematurely treatment drop-outs were captured for the weeks before, during and after the tournament. In all four periods, the admissions showed a fluctuant course.

**Fig 2:**
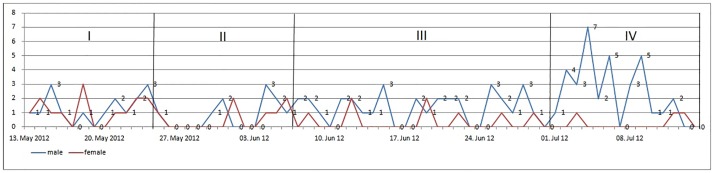
Daily admissions ahead, during and after European Football Championship 2012.

Period I to III showed similar admission patterns for both genders, while in period IV, right after the tournament, the admission rate of male patients increased significantly (p = 0.0295). The admission rate of female patients in turn decreased after the European Football Championship (Fig 2).

Average daily admission rate was calculated. In periods 2 and 3 (immediately prior and during the European Championship), the admission rate decreased compared to period 1 and 4 (Table 3).

**Table 3:** Daily admissions per period.

Period	Period duration [days]	Total admissions per day
1	13	1.8
2	13	1.0
3	24	1.2
4	13	2.0

We standardized the average admission rate for of each period and compared the distribution of admissions between the sexes. For female patients, the average admission rate did not differ between the periods (p = 0.2745). But we found a significant difference in the distribution of admissions for male patients (p = 0.0295).

**Fig 3:**
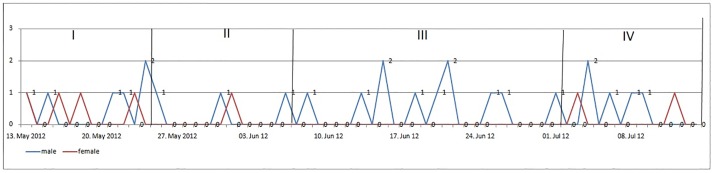
Premature drop-outs ahead, during and after European Football Championship 2012.

Also the premature treatment drop-outs were analyzed for the four periods. As the admissions, the patient drop-outs showed a fluctuant course (Fig 3).

In our study, no female patient terminated the treatment prematurely during the European Championship. For male patients, on the other hand, no change in discharge pattern could be observed. As for the admissions, daily average drop-out rate was calculated (Table 4).

**Table 4:** Daily drop-outs per period.

Period	Period duration [days]	Total discharges per day
1	13	0.8
2	13	0.5
3	24	0.5
4	13	0.5

In the first period, we found an increased average drop-out rate compared to periods 2 to 4 (Table 4). We standardized the average drop-out rate for of each period and compared the distribution of treatment drop-outs between the sexes. For male patients, the drop-out rates did not differ between the periods (p = 0.7821). For female patients, a statistically significant increase in drop-outs was found for period 1 (p = 0.0122).

## Discussion

The aim of this prospective study was to evaluate whether the European Football Championships 2012 impacted qualified detoxification treatment outcome. 125 patients from the admissions group, respectively 116 from the discharges group could be included in the study. 25.6% of the first group dropped out of detoxification treatment, whereas 30.2% of the discharges group completed the treatment irregularly. This comparatively low rate of unplanned discharges converge with data in other studies where drop-out rates between 30% and 44% for alcohol dependent patients were described [4–5]. We found an noticeable fluctuant course of admissions and drop-outs which can be explained by the fact that no patients were admitted during Pentecost (26 to 28 May 2012) and on the weekends.

We further found a significant increase in admissions of male patients after the European Football Championships had ended. These findings suggest that patients postponed detoxification treatment to not miss the tournament. It is further conceivable that previously abstinent patients relapsed in consequence of the strong association of soccer and alcohol. In first place, worldwide research demonstrated the increasingly pervasive nature of professional sports-related alcohol marketing [2]. Secondly, alcoholic beverages are commonly associated with sports [6], as for many spectators beer is a fixed component of a soccer event.

For female patients, no relation between the average admission rate and the tournament could be established.

However, no female patient terminated the treatment prematurely during the European Championship.

Our results indicate that sports events like the European Football Championships 2012 might be of less interest for female patients and therefore no motivation to prematurely terminate the treatment.

The increased drop-out rate in the first period was more likely linked to the public holidays due to Pentecost at the end of period one than to the UEFA European Football Championship. Further investigations on the influence of sports events on the likelihood of relapse shall be conducted to verify this finding, as in the present study only 32 female patients were included which limits the generalisability of the finding.

In conclusion, it is conceivable that European Football Championship 2012 impacted qualified detoxification treatment of male alcohol-dependent patients, but the present study does not allow corroboration of our finding without additional examinations of upcoming sports events. Still, one has to emphasize that statistical association does not establish causality, therefore further research could provide more accurate information if significant international sports events may serve as predictor for QDT outcome.
